# Time-Restricted Eating Regimen Differentially Affects Circulatory miRNA Expression in Older Overweight Adults

**DOI:** 10.3390/nu14091843

**Published:** 2022-04-28

**Authors:** Sunil K. Saini, Arashdeep Singh, Manisha Saini, Marta Gonzalez-Freire, Christiaan Leeuwenburgh, Stephen D. Anton

**Affiliations:** 1 All India Institute of Medical Sciences, New Delhi 110029, India; sunilsaini1403@gmail.com; 2 Department of Aging and Geriatric Research, Institute on Aging, University of Florida, Gainesville, FL 32610, USA; cleeuwen@ufl.edu; 3 Center for Integrative Cardiovascular and Metabolic Disease, Department of Pharmacodynamics, College of Pharmacy, University of Florida, Gainesville, FL 32610, USA; a.singh@ufl.edu; 4 Department of Zoology, Dronacharya Government College, Gurugram 122001, Haryana, India; manishasaini683@gmail.com; 5 Translational Research in Aging and Longevity (TRIAL) Group, Health Research Institute of the Balearic Islands (IdISBa), 07120 Palma de Mallorca, Spain; martagonzalezfreire@gmail.com; 6 Department of Clinical and Health Psychology, University of Florida, Gainesville, FL 32603, USA

**Keywords:** intermittent fasting, weight loss, cell survival, diet, fat loss

## Abstract

Time-restricted eating (TRE), a popular form of intermittent fasting, has been demonstrated to provide multiple health benefits, including an extension of healthy lifespan in preclinical models. While the specific mechanisms remain elusive, emerging research indicates that one plausible mechanism through which TRE may confer health benefits is by influencing the expression of the epigenetic modulator circulatory miRNAs, which serve as intercellular communicators and are dysregulated in metabolic disorders, such as obesity. Therefore, the goal of this pilot study is to examine the effects of a 4-week TRE regimen on global circulatory miRNA from older (≥65 years) overweight participants. Pre- and post-TRE regimen serum samples from nine individuals who participated in the Time to Eat clinical trial (NCT03590847) and had a significant weight loss (2.6 kg, *p* < 0.01) were analyzed. The expressions of 2083 human miRNAs were quantified using HTG molecular whole transcriptome miRNA assay. In silico analyses were performed to determine the target genes and biological pathways associated with differentially expressed miRNAs to predict the metabolic effects of the TRE regimen. Fourteen miRNAs were differentially expressed pre- and post-TRE regimen. Specifically, downregulated miRNA targets suggested increased expression of transcripts, including PTEN, TSC1, and ULK1, and were related to cell growth and survival. Furthermore, the targets of downregulated miRNAs were associated with Ras signaling (cell growth and proliferation), mTOR signaling (cell growth and protein synthesis), insulin signaling (glucose uptake), and autophagy (cellular homeostasis and survival). In conclusion, the TRE regimen downregulated miRNA, which, in turn, could inhibit the pathways of cell growth and activate the pathways of cell survival and might promote healthy aging. Future mechanistic studies are required to understand the functional role of the miRNAs reported in this study.

## 1. Introduction

Intermittent fasting (IF) interventions have been found to provide multiple health benefits, including combating obesity [1], insulin resistance [2], dyslipidemia [3], hypertension [4], as well as extending healthy lifespan in pre-clinical models [5]. Time-restricted eating (TRE) is a popular form of IF that restricts all calorie intake, without altering diet quantity and quality, to a 6–10 h period. Most TRE interventions have been initiated in early mornings (active phase) and have been found to provide pleiotropic metabolic health benefits, including reductions in body weight and fat, abdominal obesity, blood glucose, atherogenic lipids, and blood pressure [1,6], in clinical trials and recent meta-analyses [7,8]. 

While recent clinical studies support the feasibility of TRE in middle-aged and older adult participants [9], these studies have generally allowed participants to self-select their eating window. Such an approach may be beneficial for promoting adherence to this eating pattern; however, it is presently unclear whether the metabolic effects of TRE vary according to the time of day it is practiced (early vs. late TRE) [10]. Given the growing population of older adults with age-related metabolic disease conditions, research is needed to better understand the type of TRE that may produce the best metabolic benefits and also the type that is most acceptable to middle-age and older adults.

Although some of the mechanisms through which IF promotes beneficial metabolic effects have been described [11], the role of microRNA and their target genes in affecting these mechanisms is not well understood. Emerging studies have shown microRNA (miRNA)-mediated gene regulation as one of the mechanisms influencing lifespan in response to fasting in *C. elegans* (a model of aging) [12,13]. miRNAs are short (21–22 nucleotides), non-coding RNAs that can post-transcriptionally regulate the expression of ~60% of mammalian protein-encoding genes [14]. Recently, circulatory miRNAs are gaining interest for their potential to serve as reliable biomarkers for the diagnosis and therapeutics of numerous pathologies, including metabolic disorders [15,16,17]. These circulatory miRNAs are either present within extracellular vesicles (which includes exosomes, microvesicles, apoptotic bodies and microparticles) or are associated with protein or lipoprotein complexes [18,19]. It has been estimated that the majority of circulating miRNAs (83–99%) in serum are contained within the exosomes [20]. The discovery of miRNA in exosomes and other extracellular vesicles led to the hypothesis that they might contribute to intercellular signaling [21]. 

A growing number of animal and clinical studies have shown an association between the expression of several miRNAs in different tissues (e.g., adipose tissue, liver, and pancreas) and obesity or metabolic diseases [22,23,24]. The miRNAs secreted by the adipose tissue affect gene expression in distant organs, including the liver [22]. In obese individuals, six miRNAs (miRNA-122, miRNA-140-5p, miRNA-142-3p, miRNA-143, miRNA-222, and miRNA-486) were found to be reproducibly increased and two miRNAs (miRNA-221 and miRNA-520c-3p) to be decreased in circulation [23]. Notably, only a limited number of studies in animals and humans have investigated if there is a possible connection between calorie restriction (CR) or IF and circulatory miRNA expression. In rats, the CR-induced expression of miR-98-3p was suggested to provide neuroprotective effects and also extend a healthy lifespan [25]. In rhesus monkeys after 17 years of CR, CR-associated miRNA targets were enriched for pathways of cell growth and insulin signaling that have been implicated in delayed aging [26]. In humans, circulatory miR-500-3p and miR-770-3p, which were significantly increased with aging, were downregulated in response to short-term CR, suggesting these miRNAs as potential biomarkers of aging [27].

Metabolic disorders, including obesity and metabolic syndrome, which increase with aging, have been associated with dysregulated circulatory miRNAs. However, whether the TRE regimen affects the expression of circulatory miRNAs that serve as intercellular communicators and can regulate biological pathways to provide metabolic benefits remains unknown. Understanding the molecular mechanisms that produce beneficial effects of TRE in older adults is therefore of great significance to predict novel intervention targets. 

In this pilot study, we adopt an unbiased approach to profile human circulatory miRNAs and determine whether circulatory miRNAs are differentially expressed in the same participants pre- and post-TRE regimen. We also performed in silico pathway analyses of differentially expressed miRNA targets genes to determine the associated biological pathways in response to a 4-week TRE intervention. Knowing what miRNAs change in circulation in response to TRE could aid in identifying potential miRNA targets to develop novel pharmacological therapeutics that can benefit people who cannot fast or are not willing to fast. 

## 2. Methods

### 2.1. Participants Recruitment

Participants were recruited as described previously [9]. Briefly, 9 overweight older adults (6 Females, 3 Males; aged 65 years and older), who had mild to moderate functional limitations, were recruited to participate in the Time to Eat pilot clinical trial (NCT03590847). 

### 2.2. Intervention

All participants were advised to fast for approximately 16 h per day for four weeks with the daily target range set for 14–18 h. Participants were asked to abstain from any caloric intake during the targeted fasting window of 16 continuous hours. There were no dietary restrictions on the amount or types of food consumed during the 8 h eating window, and participants were allowed to choose a time frame that best fit their lifestyle. Participants were encouraged to hydrate during fasting times. Notably, participants self-selected their fasting and eating time periods, with the vast majority of participants choosing to fast between the hours of 7 p.m. to 10 a.m.

### 2.3. Sample Collection

Blood was collected from all participants pre- and post-TRE regimen in the mornings. At both time points, blood was collected in a fasted state (14–16 h fast). Blood was drawn into serum tubes, inverted five times, and allowed 30–60 min clotting time at room temperature. Tubes were centrifuged at 1600× *g* for 15 min at 4 °C, serum aliquoted, and then immediately stored in a −80 °C freezer. 

### 2.4. miRNA Expression Profiling and Analyses

Serum was used to profile the expression of circulatory miRNA using HTG (HTG Molecular Diagnostics, Inc., Tucson, AZ, USA) EdgeSeq miRNA Whole Transcriptome Assay [28]. Briefly, the assay measured the expression of 2083 human miRNA transcripts using next-generation sequencing. The assay was performed at HTG Molecular lab (Tucson, AZ, USA). HTG internal work instructions and operating procedures were used to conduct the experiment, data processing, and expression counts for each miRNA. A list of procedures can be provided upon request. Data were analyzed for miRNA differential expression between pre-and post-TRE regimen using DESeq2 package. miRNAs with very low read counts (i.e., read count < 10) were excluded from the analyses. 

### 2.5. Bioinformatic Analysis

The miRNA targets were identified using a target mining tool from miRWalk Version 3 [29], which utilizes a random-forest-based approach to integrate six conventional features and seven new features to predict miRNA target sites [30]. Putative targets were identified by selecting Target Scan and miRDB target filter and a score of <0.95 for binding energy. Gene Set enrichment analysis (GSEA) was performed to identify significantly enriched Kyoto Encyclopedia of Genes and Genomes (KEGG) pathways and Gene Ontology term (Biological Process, Molecular Function and Cellular Process) associated with miRNA targets. Significance for KEGG pathway and Gene Ontology term was considered with an adjusted (Benjamini–Hochberg adjusted) *p*-value < 0.05. Putative target proteins were assessed for protein–protein interactions (PPIs) using STRING protein–protein network analysis [31]. K-means clustering was performed on the network to highlight major clusters [32]. Clusters with protein–protein interactions (PPIs) with a *p*-value < 0.05 were considered statistically significant.

### 2.6. Statistical Analyses 

For statistical analyses, a paired *t*-test was performed on data collected from the same participants pre- and post-TRE regimen and miRNAs with a *p*-value < 0.05 were considered significantly different. 

## 3. Results

### 3.1. Identification of Differentially Expressed miRNAs

**Figure 1:**
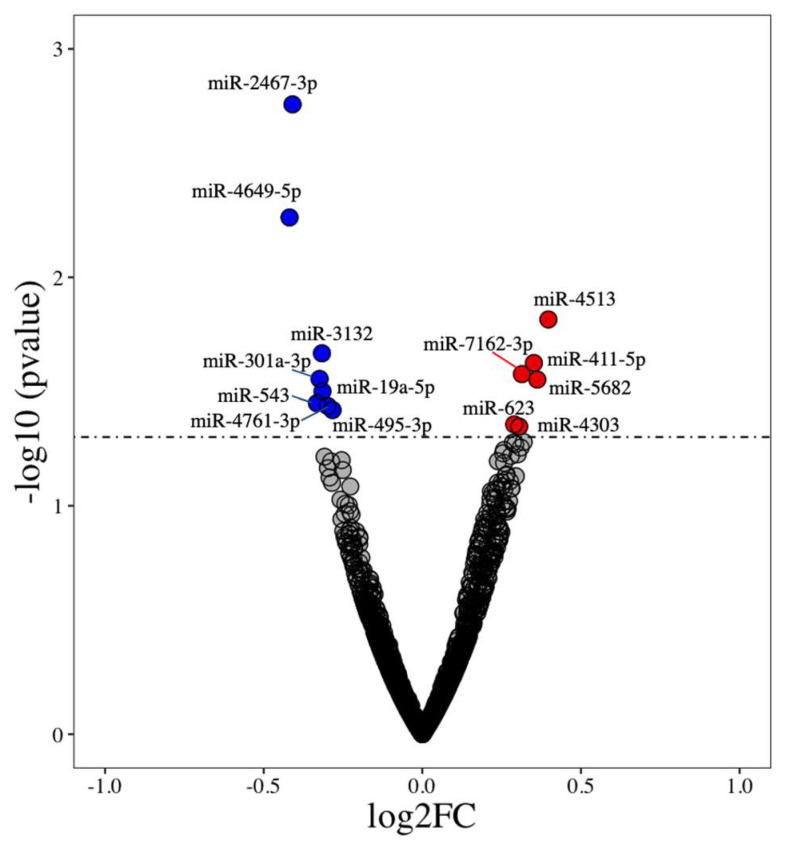
Differentially expressed miRNAs between human subjects during the pre- and post-TRE regimen. Highlighted are the names of differentially expressed miRNAs with *p* < 0.05. Upregulated miRNAs are represented on the upper right quadrant (red dots) and downregulated miRNAs on the upper left quadrant (blue dots).

Of 2083 human miRNA transcripts, there were significant differences in the expression of 14 miRNAs (*p* < 0.05) in the same participants pre- and post-TRE regimen [9] (Table 1). Among these, eight were downregulated (miR-4649-5p, miR-2467-3p, miR-543, miR-301a-3p, miR-3132, miR-19a-5p, miR-495-3p, and miR-4761-3p) and six were upregulated (miR-623, miR-4303, miR-7162-3p, miR-411-5p, miR-5682, and miR-4513) (Figure 1). The complete list of miRNAs, their fold change, and *p*-values are provided in Supplementary Materials file S1.

### 3.2. miRNA Target Genes and Pathways

All differentially expressed miRNAs were evaluated for potential targets using the miRWalk target mining tool. Interestingly, we found 127 potential targets for the 8 downregulated miRNAs (Supplementary Materials file S2). Gene set enrichment analysis (GSEA) was performed for the potential targets to identify significantly associated pathways and gene ontology term. A total of 17 KEGG pathways related to cell proliferation, cell signaling, differentiation, and cell survival, were identified as significant biological pathways (Table 2). The top pathways included the ErbB signaling pathway, Ras signaling pathway, mTOR signaling pathway, insulin signaling pathway, and autophagy. For the six upregulated miRNAs, no potential target was identified.

### 3.3. miRNA Target Interaction

**Figure 2:**
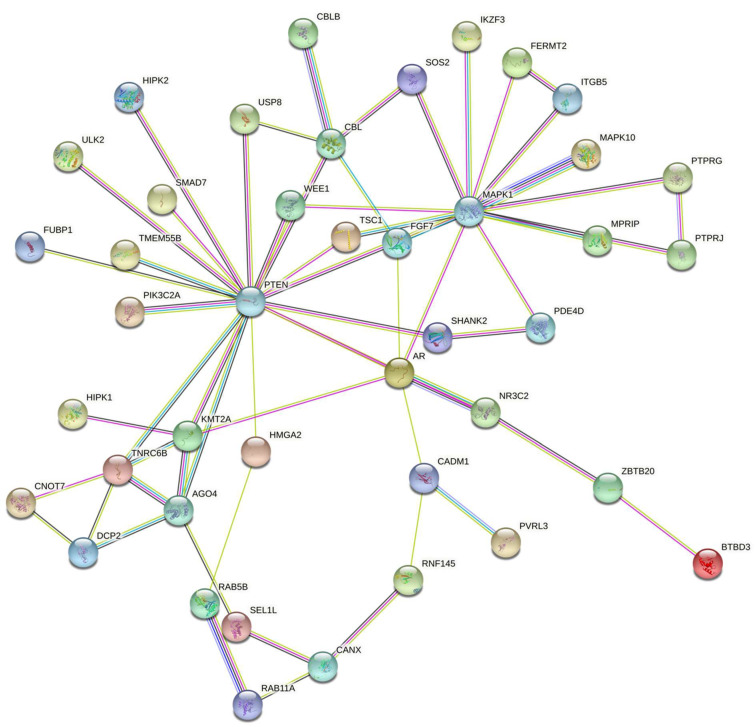
Protein–protein interaction analysis representing the interaction networks of miRNA target proteins, depicting two major clusters (PTEN and MAPK1) in the network.

To understand the regulatory network of protein targets of downregulated miRNAs, the STRING database was used to perform protein–protein interaction (PPI) network analysis. The network analysis yields a significant PPI score (PPI enrichment *p* = 6.03 × 10^−7^) with two major clusters (PTEN and MAPK1) represented by transcription factors that could act as upstream regulators to stimulate or repress gene expression (Figure 2). The proteins associated with the PTEN cluster included PIK3C2A, NR3C2, MAPK10, TSC1, TMEM55B, TNRC6B, and AGO4. The proteins associated with the MAPK1 cluster were MPRIP, TSC1, FGF7, IKZF3, FERMT2, AR, and PTEN. Taken together, the downregulated miRNAs could directly or indirectly target and regulate the expression of the PTEN and MAPK1 transcription factors and their downstream effector genes, respectively, to influence the associated biological pathways (Table 2).

## 4. Discussion

In this study, we aimed to identify circulatory miRNAs that were differentially expressed after a TRE intervention in older, overweight adults. The key findings of this study are that 14 miRNAs were differentially expressed (8 downregulated and 6 upregulated) in participants in response to the TRE regimen. Furthermore, the enrichment analysis of the targets of downregulated miRNAs indicated TRE leads to the modulation of pathways related to: the ErbB signaling pathway, Ras signaling pathway, mTOR signaling pathway, insulin signaling pathway, and autophagy. Interestingly, the targets of downregulated miRNAs were associated with both (1) growth signaling pathways, i.e., Ras signaling and mTOR signaling; and (2) repair/cell survival signaling pathways, i.e., insulin signaling and autophagy. The identified differentially expressed miRNAs in our study were mostly novel and remain rarely documented in the literature, largely because previous studies have analyzed only a targeted subpopulation of miRNAs, thus limiting the ability to discover novel miRNAs. Taken together, our findings suggest that the TRE regimen can modulate the expression of miRNAs that are involved in regulating the expression of transcripts related to cell growth and cell survival/metabolic adaptations and, thus, can be a promising therapeutic tool for aging. 

Our study adopted an unbiased approach to profile human circulatory miRNAs to evaluate whether circulatory miRNAs are differentially expressed among the same participants pre- and post-TRE regimen. Recently, several miRNAs have been identified to regulate adipose tissue biology, insulin secretion, and action in the development of obesity and related metabolic complications [17]. For instance, miR-14, miR-278, and let-7 have been reported to be involved in controlling lipid and glucose metabolism [17]. Notably, miR-2467-3p and miR-4649-5p, the most downregulated and significant miRNAs in our study, have been recently reported as a potential marker for gestational diabetes mellitus [33] and as a regulator of lipid metabolism, respectively [34]. Furthermore, miR-543 and miR-301, commonly upregulated miRNAs in cancer [35,36] and upregulated in type 2 diabetes [36], were downregulated after the TRE intervention in our study. 

Similar to the TRE, Ramadan fasting where the time period of feeding is restricted (14 h daily fasting period) has been found to provide positive health benefits [37] and is associated with the downregulating expression of biomarkers of obesity in humans [38,39]. Furthermore, meta-analyses also suggest that Ramadan fasting could aid in reducing low-grade systemic inflammation and oxidative stress [40]. Our findings on miRNAs and their targets in conjunction with recent clinical studies using Ramadan fasting [39] highlight that TRE could promote beneficial effects on gene expression related to aging and metabolism; however, additional studies are warranted to deduce causality. Taken together, miRNAs could be differentially expressed with interventions, such as TRE, and the regulation of the expression of genes and pathways to produce beneficial effects, highlighting their therapeutic potential [41]. 

Using a combination of miRNA target prediction algorithms (highly predicted and experimentally validated targets), the downregulated miRNAs yielded 127 targets. These targets included transcripts involved in metabolic and longevity pathways, such as Ras signaling pathway, mTOR signaling pathway, insulin signaling pathway, and autophagy. The transcripts commonly involved in the above-mentioned pathways included PTEN, TSC1, ULK2, MAPK10, and MAPK1. PTEN is a protein and lipid phosphatase that removes the phosphate in tyrosine-, serine-, and threonine-kinases, and phosphatidylinositol, respectively. PTEN phosphatase activity negatively regulates the PI3K-AKT/PKB and mTOR signaling pathway and, thus, promotes cell survival over cell growth [42]. PTEN also negatively regulates insulin signaling and glucose metabolism in adipose tissue [42]. TSC1 gene encodes growth inhibitory protein hamartin, which negatively regulates mammalian target of rapamycin complex 1 (mTORC1) signaling. TSC1 forms a complex with TSC2 and inhibits the nutrient-mediated or growth-factor-stimulated phosphorylation of S6K1 and EIF4EBP1 by negatively regulating mTORC1 signaling [43,44]. ULK1 is a serine/threonine-protein kinase involved in autophagy in response to starvation [45]. ULK1 acts upstream of phosphatidylinositol 3-kinase (PIK3C3) to regulate the formation of autophagophores, the precursors of autophagosomes. It also acts both as a downstream effector and a negative regulator of the mammalian target of rapamycin complex 1 (mTORC1). mTOR signaling is known to positively regulate cell growth and protein synthesis pathways. Together, these targets, while participating in nutrient-sensing pathways, were suggested to inhibit mTOR signaling, thus inhibiting protein synthesis pathway, and enhance autophagy, an important process that drives cell survival. The suppression of mTOR signaling and increase in autophagy have been suggested to contribute to many of the beneficial adaptations seen with IF protocols in clinical populations [6,46]. Since miRNA downregulates gene expression, the downregulation of a miRNA will ultimately result in the upregulation of its target. Collectively, these results suggest that the downregulation of miRNA in response to TRE might result in an increased expression of transcripts, such as PTEN, TSC1, and ULK1, which in turn could inhibit the pathways of protein synthesis/cell growth and activate the pathways of cell survival/metabolic adaptations, thus promoting healthy aging.

This pilot study is important as it compared the circulatory miRNAs within the same subjects that were subjected to the TRE regimen, but has a few limitations. Firstly, the circulatory miRNAs that were identified as differentially expressed were at a nominal *p*-value (*p* < 0.05) and not adjusted for multiple comparisons. The sample size was small and therefore could have limited the statistical power to detect significant differences in miRNA expression. Thus, these initial findings need to be validated in larger cohorts before generalizability can be inferred. Secondly, the study was limited to the identification of potential biological pathways affected by miRNA expression in participants post-TRE regimen; and whether these pathways are functionally activated or inhibited in humans requires further validation. Hence, the findings reported in this pilot study should be considered exploratory. Thirdly, the experimental validation of miRNA targets was not performed. Fourthly, caloric and dietary intakes have a major impact in determining the magnitude of changes in epigenetic modification; therefore, more comprehensive assessment and holistic examination are needed to increase our understanding of the factors that may influence miRNA changes upon TRE. Taken together, given the small sample size and the observational nature of these analyses, no causal inferences can be made from this study.

## 5. Conclusions

The findings of this pilot study unraveled 14 circulatory miRNAs, which were differentially expressed in participants pre and post to the TRE regimen. The downregulated miRNA targets suggested an increased expression of transcripts, such as PTEN, TSC1, and ULK1, which in turn could inhibit the pathways of cell growth and activate the pathways of cell survival and might promote healthy aging. The results of our study warrant a further investigation of the potential health-promoting effects of TRE on the identified miRNAs in larger samples over longer time periods. 

## Figures and Tables

**Table 1:** List of differentially expressed miRNAs in participants post-TRE regimen.

miRNA Name	log2 Fold Change	*p*-Value
miR-2467-3p	−0.41	0.002
miR-4649-5p	−0.42	0.005
miR-4513	0.40	0.015
miR-3132	−0.32	0.021
miR-411-5p	0.35	0.024
miR-7162-3p	0.31	0.027
miR-301a-3p	−0.32	0.028
miR-5682	0.36	0.028
miR-19a-5p	−0.31	0.032
miR-543	−0.33	0.036
miR-495-3p	−0.30	0.037
miR-4761-3p	−0.28	0.038
miR-623	0.29	0.044
miR-4303	0.31	0.045

**Table 2:** Associated biological pathways with miRNA targets: KEGG pathway.

Name	Genes	Adjusted *p*-Value
ErbB_signaling_pathway	CAMK2G; MAPK10; CBLB; MAPK1; CBL; SOS2	0.002
Ras_signaling_pathway	GRIN2A; RAB5B; MAPK10; FGF7; MAPK1; RALBP1; RASGRF2; SOS2	0.006
mTOR_signaling_pathway	PTEN; TSC1; ULK2; LRP6; MAPK1; SOS2	0.006
Insulin_signaling_pathway	TSC1; MAPK10; CBLB; MAPK1; CBL; SOS2	0.006
Pathways_in_cancer	AR; PTEN; ARHGEF12; CAMK2G; MAPK10; FGF7; LRP6; MAPK1; CBL; RALBP1; SKP1; SOS2	0.006
cAMP_signaling_pathway	GRIN2A; PDE3A; PDE4D; CAMK2G; MAPK10; MAPK1	0.014
Autophagy	PTEN; TSC1; ULK2; MAPK10; MAPK1	0.014
Regulation_of_actin_cytoskeleton	ENAH; ARHGEF12; ITGB5; FGF7; MAPK1; SOS2	0.014
Proteoglycans_in_cancer	ARHGEF12; CAMK2G; ITGB5; MAPK1; CBL; SOS2	0.014
Breast_cancer	PTEN; FGF7; LRP6; MAPK1; SOS2	0.014
Cellular_senescence	PTEN; TSC1; HIPK2; HIPK1; MAPK1	0.0153
Tuberculosis	ARHGEF12; CAMK2G; RAB5B; MAPK10; MAPK1	0.0218
Focal_adhesion	PTEN; MAPK10; ITGB5; MAPK1; SOS2	0.0294
Salmonella_infection	RAB5B; MAPK10; MAPK1; SKP1; GCC2	0.0332
Human_immunodeficiency_virus_1_infection	TNFRSF1B; WEE1; MAPK10; MAPK1; SKP1	0.0332

## Data Availability

All the data is provided in the supplementary files.

## Supplementary Materials

The following supporting information can be downloaded at: https://www.mdpi.com/article/10.3390/nu14091843/s1; Supplementary Materials file S1: results; Supplementary Materials file S2: miRWalk_miRNA_Targets.
